# Coffee Consumption and the Incidence of Colorectal Cancer in Women

**DOI:** 10.1155/2016/6918431

**Published:** 2016-04-28

**Authors:** Erik J. Groessl, Matthew A. Allison, Joseph C. Larson, Samuel B. Ho, Linda G. Snetslaar, Dorothy S. Lane, Katie M. Tharp, Marcia L. Stefanick

**Affiliations:** ^1^University of California, San Diego, La Jolla, CA, USA; ^2^VA San Diego Healthcare System, San Diego, CA, USA; ^3^Fred Hutchinson Cancer Research Center, Seattle, WA, USA; ^4^University of Iowa, Iowa City, IA, USA; ^5^Stony Brook University, Stony Brook, NY, USA; ^6^Loras College, Dubuque, IA, USA; ^7^Stanford University, Stanford, CA, USA

## Abstract

*Background.* Higher coffee consumption has been associated with decreased incidence of colorectal cancer. Our objective was to examine the relationship of coffee intake to colorectal cancer incidence in a large observational cohort of postmenopausal US women.* Methods.* Data were collected for the Women's Health Initiative Observational Study providing a follow-up period of 12.9 years. The mean age of our sample (*N* = 83,778 women) was 63.5 years. Daily coffee intake was grouped into 3 categories: None, moderate (>0–<4 cups), and high (4+ cups). Proportional hazards modeling was used to evaluate the relationship between coffee intake and colorectal cancer.* Results.* There were 1,282 (1.53%) new cases of colorectal cancer during follow-up. Compared to nondrinkers, moderate and high coffee drinkers had an increased incidence of colorectal cancer in multivariate analysis (HR 1.15, 1.02–1.29; HR 1.14, 0.93–1.38). Moderate drip brew coffee intake (HR 1.20, 1.05–1.36) and high nondrip brew coffee intake (HR 1.43, 1.01–2.02) were associated with increased odds.* Conclusion.* Our results suggesting increased incidence of colorectal cancer associated with higher coffee consumption contradict recent meta-analyses but agree with a number of other studies showing that coffee increases risk or has no effect. Brew method results are novel and warrant further research.

## 1. Introduction

The potential links of coffee consumption to various health conditions including cancer have been studied extensively. While most early studies were concerned that coffee consumption was carcinogenic and would lead to higher rates of cancers, subsequent efforts to control for other factors and comprehensive review studies found that although many case-control studies linked coffee consumption to increased rates of cancer, multiple larger prospective trials found no connection [1–4]. After many additional studies, it was concluded in 2008 that there is a “convincing” level of evidence that there is no relationship between coffee intake and breast cancer, and a “probable” level of evidence that there is no relationship between coffee intake and pancreatic cancer, ovarian cancer, thyroid cancer, prostate cancer, bladder cancer, bone health, cardiovascular disease, and reproductive adversities [5]. However, for a number of other cancers, including colorectal cancer, the evidence of a relationship with coffee is rated “possible,” suggesting further research is warranted [5].

Most of the accumulated evidence for colorectal cancer indicates that higher daily coffee consumption is associated with* reduced* incidence of colorectal cancer [5–7]. The most recent meta-analysis of the association between coffee consumption and colorectal cancer conducted separate analyses for case-control evidence and cohort study evidence and found that when comparing the highest category of coffee consumption to the lowest category, high coffee consumption was associated with a significantly reduced risk of colorectal cancer (OR = 0.85, 95% CI 0.75, 0.97) [6]. When combining 16 cohort studies, high coffee consumption trended towards a slight reduction in the risk of colorectal cancer (OR = 0.94, 95% CI 0.88, 1.01). Contrary to the above findings, which found the strongest overall relationship between coffee and colon cancer, a 2010 study that only focused on prospective cohort studies found no relationship between coffee consumption and risk of colon cancer [8]. Since the meta-analyses described above, data from three large prospective cohort studies have explored this question, with one study finding a reduced risk of colorectal cancer for those with high coffee intake, one study finding no significant differences [9], and the third finding coffee to be associated with an increased risk [10].

Thus, although most studies and meta-analyses have concluded there is some evidence of a negative relationship between coffee consumption and colorectal cancer, conflicting evidence from a well-designed meta-analysis [8] and recent large cohort studies highlights the need for additional research in this area [9]. All of the recent studies and meta-analyses state that the relationship remains unclear [5–10], and evidence suggesting a stronger protective effect among women should be explored. Our current objective is to examine the relationship of daily consumption of caffeinated and decaffeinated coffee to colorectal cancer in a large observational cohort of older women in the United States (US). Hypothesized mechanisms such as phenolic phytochemicals cannot be explored in this study, but caffeinated versus decaffeinated coffee will be studied, along with coffee preparation methods that may impact the amount of fiber reaching the gastrointestinal tract [11].

## 2. Methods

### 2.1. Study Population

The Women's Health Initiative is a large, ongoing national health study of women in the US aged 50–79 at inception. The Women's Health Initiative consisted of three overlapping clinical trials and an observational study. Since the original study period, two extensions have allowed for ongoing collection of follow-up data from consenting participants. The current study uses data from the Women's Health Initiative Observational Study (WHI OS) and the follow-up extensions.

The WHI OS design and measures have been described in detail in previous publications [12, 13]. In summary, 93,676 women between the ages of 50–79 were recruited from October 1993 through December 1998 at 40 clinical recruitment centers regionally distributed across the US. To be eligible, participants also had to be postmenopausal and be unlikely to die or move in the next 3 years. From the original 93,676, we excluded 959 participants who had a history of colorectal cancer at baseline and an additional 773 participants with missing coffee data. Finally, we removed 8,166 participants with missing model covariate data and/or no follow-up time, giving a final analysis sample of 83,778. Among this sample the average follow-up time was 12.9 years.

### 2.2. Measures

Demographic data, lifestyle, and medical history were collected using self-report questionnaires at a baseline visit for all enrolled participants. Physical measurements such as height and weight were collected by trained staff at a baseline clinical visit. Health outcomes data were collected annually by mailed questionnaires.

Coffee intake was measured at baseline by a question asking “How many cups of regular coffee (not decaf) do you usually drink each day? (Count tall [12 ounces. or more] cups and espresso drinks made with double shots of espresso as 2 cups.)” Response categories were “None”; “1 cup”; “2-3 cups”; “4-5 cups”; “6 or more cups.” Decaffeinated coffee was measured the same way, replacing the word “regular” with “decaffeinated.” Tea consumption was measured with the same type of question but referred only to caffeinated tea. Other dietary variables were derived from a food frequency questionnaire [14].

Colorectal cancer history was measured at baseline using a comprehensive medical history questionnaire. Medical history updates were conducted annually thereafter. Any indication of possible colorectal cancer was verified by a review of medical records by trained physician adjudicators at each site before being confirmed at the WHI clinical coordinating center via blinded review. More details on the identification of historical and incidental colorectal cancer cases are provided in previous publications [15]. The frequency of bowel screening was not standardized as part of the WHI OS. Information on various types of bowel screening was collected by self-report questionnaire during annual assessments.

### 2.3. Statistical Analysis

Baseline characteristics of the sample by coffee intake (Table 1) are presented with means with standard deviations for continuous variables and frequencies with percentages for categorical variables. Statistical comparisons between groups are done with *t* (continuous variables) and chi-square (categorical variables) tests for differences by colorectal cancer. Differences in linear trends of coffee drinkers by demographic characteristics were assessed by fitting either a linear (continuous/ordinal variables) or logistic (dichotomous variables) model with the demographic variable as a function of linear coffee (0 cups/day = 1; >0–<4 cups/day = 2; ≥4 cups/day = 3).

**Figure 1:**
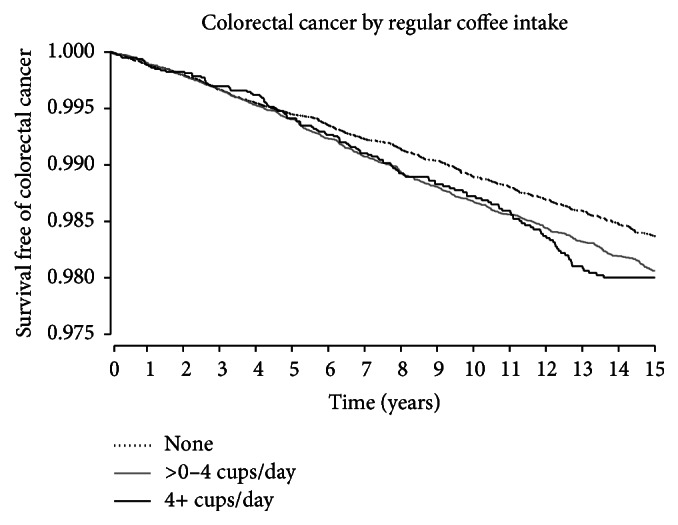
Colorectal cancer survival by coffee intake.

Proportional hazards modeling was used to evaluate the relationship between coffee variables and colorectal cancer (Table 2), following participants from enrollment in the WHI observational study until they either had an event, died, or were lost to follow-up. Prior to modeling, the proportional hazards assumption was checked by fitting models with each colorectal cancer outcome as a function of coffee intake and its interaction with the log survival time. Modeling was done in two ways, first with each outcome as a function of regular coffee intake unadjusted, then adjusted for age, ethnicity, education, alcohol use, smoking/pack-years, BMI, physical activity, total energy intake, red meat intake (oz.), fruit/vegetable intake (cups), percent caloric intake from fat, fiber intake (g), calcium intake (diet plus supplements, mg), hormone use (with type if current), NSAID use, history of treated diabetes, and family history of colorectal cancer. Hazard ratios and their corresponding confidence intervals are presented for each outcome. Separate models were then used to create accompanying linear trend *p* values, substituting the categorical coffee variable for a linear version (0 cups/day = 1; >0–<4 cups/day = 2; ≥4 cups/day = 3) with *p* values for the linear coffee term presented. To examine whether results may differ for decaffeinated and total coffee consumption, we ran sensitivity analyses substituting each for regular caffeinated coffee in our primary models (Appendix Tables A and B in Supplementary Material available online at http://dx.doi.org/10.1155/2016/6918431). In addition, the questionnaires contained one question about consumption of caffeinated tea only. Caffeinated tea consumption was also tested as a possible covariate. To examine effects of adjustment variables on colorectal cancer, full model results of the three-level regular coffee variable are also included (Appendix Table C). To get a graphical look at colorectal cancer survival by coffee groups, survival curves were constructed (Figure 1).

In addition to the primary models, additional models were run to determine whether the effect of coffee on colorectal cancer varied by subgroup variables of age, ethnicity, alcohol use, smoking/pack-years, HT use, HT type (among those using HT), BMI, and coffee type. For each subgroup a proportional hazards model was fit modeling colorectal cancer as a function of coffee intake, the subgroup of interest, their interaction, and all adjustment variables used in the primary models. As with the primary models, *p* values are calculated from separate trend models. All analyses were conducted using SAS for Windows version 9.4.

## 3. Results

Complete data for the WHI OS sample is available in previous publications [13]. Characteristics of our sample are presented by colorectal cancer status Table 1. During the mean follow-up of 12.9 years, 1,282 (1.53%) participants had documented cases of colorectal cancer. Older age, higher BMI, less physical activity, higher fat intake, not using hormone replacement therapy, a history of treated diabetes, and a family history of colorectal cancer were all significantly associated with incident colorectal cancer.

Characteristics of our sample are also presented by level of coffee consumption in Table 2. Participants with higher coffee consumption tended to be slightly younger, were more likely to be White, and were less likely to have a college degree. They also were more likely to be a current smoker and/or alcohol drinker and have a higher BMI. Many of the effects were quite small but were statistically significant.

Prior to modeling we checked the proportional hazards assumption and found no significant interactions between the primary coffee variable and the log survival time for any of our outcomes, indicating that the proportional hazards assumption has not been violated in our models. In unadjusted models, we explored whether the relationship of coffee consumption with colorectal cancer differed when including or removing decaffeinated coffee from the consumption variable. We found that including decaffeinated coffee had a negligible effect on results. Thus, the consumption variable in the final models included only regular (caffeinated) coffee. We also examined caffeinated tea consumption as a covariate. Very few study participants were regular tea drinkers and this variable was not significantly related to cancer incidence or coffee consumption.

Three-level regular coffee consumption was associated with colorectal cancer, with results for both low and high coffee intake relative to non-coffee drinkers (HR 1.18, 95% CI 1.05–1.33; HR 1.22, 1.00–1.47, resp.) (see Table 2). After multivariate adjustment, this association remained significant (*p* = 0.04) but the confidence interval for 4+ cups per day was of borderline nonsignificance. In the multivariate model for 3-level coffee consumption (see Appendix Table C), significant predictors of colorectal cancer included age (HR 1.32, 95% CI 1.26–1.37 for a 5-year increment), education (HR 1.26, 1.08–1.47; and HR 1.23, 1.05–1.44 for higher education groups versus ≤high school/GED), smoking (HR 1.34, 1.15–1.56 for past, ≥20 pack-years versus never smokers; HR 1.63, 1.26–2.11 for current, ≥20 pack-years versus never smokers), BMI (HR 1.10, 1.05–1.15 for a 5 kg/m^2^ increase), current hormone use (HR 0.72, 0.62–0.84 for E-Alone versus never, and HR 0.75, 0.64–0.89 for E + P versus never), current NSAID use (HR 0.87, 0.77–0.97), and a positive family history of colorectal cancer (HR 1.24, 1.08–1.43).

We also examined the relationship between coffee consumption and the three main types of colorectal cancer (Table 2). Like all colorectal cancers together, colon cancer occurred more often among those who drank 4+ cups of coffee per day. However, this effect was no longer significant after adjusting for covariates.

The interaction effects of coffee consumption levels by covariate groups (age, ethnicity, alcohol use, smoking, current HT use, current HT type, BMI, and coffee brew type) on colorectal cancer were examined and only coffee brew type was borderline nonsignificant (*p* = 0.06). Participants who primarily consumed drip coffee had relatively stable hazard ratios relative to non-coffee drinkers regardless of the amount of consumption. Non-drip coffee drinkers, however, had increased hazard ratio for colorectal cancer relative to non-coffee drinkers as coffee consumption increased. Details are available in Appendix Table D.

## 4. Discussion

The results of our unadjusted model suggest that drinking regular caffeinated coffee, both in moderate amounts (1–3 cups/day) or in larger amounts (4+ cups/day) was associated with an increased risk of colorectal cancer in postmenopausal women. After controlling for covariates in the multivariate model, only the group reporting coffee consumption between 0 and 4 cups per day remained statistically significant, while the group reporting more than four cups per day was of borderline significance, despite the large sample size.

This finding contrasts with other literatures on the association of coffee consumption with colorectal cancer, which suggests a protective effect of coffee, especially in larger amounts such as 4-5 or more cups daily [5–7].

The most recent meta-analysis for the association between coffee consumption and colorectal cancer conducted separate analyses for case-control evidence and cohort study evidence and distinguished between colorectal cancer, colon cancer, and rectal cancer [6]. When combining the results of 25 case-control studies, they found that when comparing the highest category of coffee consumption to the lowest category, high coffee consumption was associated with a significantly reduced risk of colorectal cancer (Odds Ratio (OR) = 0.85, 95% CI 0.75, 0.97) and colon cancer (OR = 0.79, 95% CI 0.67, 0.95) but not rectal cancer alone (OR = 0.95, 95% CI 0.79, 1.15). When combining 16 cohort studies, high coffee consumption trended towards a slight reduction in the risk of colorectal cancer (OR = 0.94, 95% CI 0.88, 1.01) and colon cancer (OR = 0.93, 95% CI 0.86, 1.01) but not rectal cancer alone (OR = 0.98, 95% CI 0.88, 1.09). Subgroup analyses in the case-control studies indicated that the relationship between coffee consumption and reduced risk of colorectal cancer was significant among women and Europeans while the relationship between coffee consumption and reduced risk of colon cancer was significant among Europeans. However, in the cohort studies, the relationship between coffee consumption and reduced risk of colon cancer was significant only for Asian women. Contrary to the above findings, which found the strongest overall relationship between coffee and colon cancer, a 2010 study that only focused on prospective cohort studies found no relationship between coffee consumption and risk of colon cancer [8].

Since the meta-analyses described above, data from three large cohort studies have explored this question. In 2012, data from a large prospective study of older adults [7] with a mean follow-up of 10.5 years showed that when compared to coffee nondrinkers, there was a reduced risk of colon cancer among people who drank 4-5 cups of coffee daily (HR = 0.85, 95% CI: 0.75, 0.96) and those who drank 6 or more cups daily (HR = 0.74, 95% CI: 0.61, 0.89). When broken down by caffeinated and noncaffeinated coffee, results for drinkers of caffeinated coffee were similar to the overall results, while the effect was smaller and only trended towards significance for drinkers of decaffeinated coffee. In 2013, a large prospective cohort study of 57,398 men and women in the US did not find any significant associations between coffee intake and risk of colorectal cancers, despite exploring many subgroups and variables that have been significant elsewhere [9]. Finally, in 2014, a large prospective cohort study of 58,221 Japanese men and women found an increased risk of colon cancer for men drinking 2-3 cups per day (hazard ratio (HR) = 1.26) and men drinking 4 or more cups per day (HR 1.79) [10]. No effect was found for women, but very few Japanese women in this study were coffee drinkers.

In summary, although the majority of studies have found a protective effect of higher coffee intake with respect to colorectal cancer, a number of recent high quality studies have found no effect [8, 9, 16], while others found an increased risk of cancer as was found in our current study of older women [10]. Our study is different from most others in that it focuses only on women.

When breaking all colorectal cancers into the subtypes of colon, rectal, and rectosigmoid, we did not find any significant relationship between regular coffee consumption. With a total of 1,282 participants with colorectal cancer identified, dividing those into cancer subgroups and then examining by 5 groups of coffee consumption by brew method resulted in subgroups with small sample sizes, limiting statistical power. Despite this limitation, there was some indication or trend towards a higher incidence of colon cancer in participants drinking more than four cups of coffee and using nondrip brew methods. Like our primary findings, this contradicts recent meta-analytic results which suggest a possible protective effect of higher coffee consumption for incident colon cancer [6].

Multiple mechanisms by which coffee (both caffeinated and decaffeinated) may impact colorectal cancer have been postulated and studied, but few firm conclusions have emerged [17]. Although many research studies focus mainly on caffeine content, coffee contains over a thousand different chemical compounds including phenols, melanoidins, and diterpenes that have antioxidant effects and may have health benefits [18]. Coffee contains larger amounts of phenolic phytochemicals than either red wine or tea. One recent study showed that caffeic acid and not caffeine suppressed cancer cell growth in colon cancer [17]. Other research concludes that protective effects of coffee against colorectal cancers are likely due to the high concentrations of polyphenols and dietary fiber that reach the colon [18]. One of the unique contributions of this study is that we were able to include information on the method by which coffee was brewed in our analyses. Very few other analyses include this information, and our results show that the brewing method for coffee was related to incidence of colorectal cancers. Specifically, drinkers of more than four cups of coffee per day who did not use drip coffee methods were more likely to get colorectal cancer. This contradicts the potential mechanisms proposed by some researchers [18], who posited that because nondrip filters allow more dietary fiber and potentially antioxidants to be released into the coffee [11], colon motility may be increased, potentially resulting in reduced risk of colorectal cancers. Since Europeans use the drip method and paper filters less often than in the United States, the finding of reduced risk among Europeans was seen as possible support for this theory [18]. Our data suggest that it is important for future research to study the method of coffee preparation when examining the relationship between coffee consumption and colorectal cancers.

The most recent research in this area has identified over 20 biomarkers associated with coffee consumption in the Prostate, Lung, Colorectal, and Ovarian (PLCO) Cancer Screening Trial Study [19]. Some of these biomarkers have been linked to reduced rates of colorectal cancer, and are thus promising targets for research on the mechanisms by which coffee might affect colorectal cancers. Another recently published study of colorectal cancer recurrence found that Japanese men drinking 3 or more cups of coffee daily had reduced recurrence of proximal colorectal cancer, yet coffee consumption was also associated with increased incidence of distal colorectal cancer recurrence [20]. Thus, the continued study of the different subtypes of colorectal cancer is warranted.

A strength of our study is that any indication of cancer was followed up by a review of medical records by study physicians and adjudicators. Although the study was not designed to produce a nationally representative sample, WHI had 40 clinical research centers recruiting in 24 states across the US and was able to enroll large numbers of women from groups that typically are harder to recruit, minority women and women aged 70–79 [21]. While no other studies focused exclusively on women, the incidence of cancer in our study (0.118% annually) seemed quite comparable to the incidence (0.136% annually) in another large prospective study that also included men [7]. Conversely, a possible limitation is that the participant pool is not fully representative of all women but was limited to postmenopausal women, the age at which colorectal cancer is most prevalent. The study was limited by the questions asked in the study questionnaires. The questions grouped coffee consumption into 5 categories (“None”; “1 cup”; “2-3 cups”; “4-5 cups”; “6 or more cups”) instead of using a continuous scale. The questionnaire did not ask about herbal tea usage, which is also frequently studied in published analyses similar to ours. It is also possible that there are other confounding factors that were not measured or omitted from analyses. One such possible factor, aspirin use, has been linked to incidence of colorectal cancer in the past [22], but a prior study with the WHI cohort found no relationship [12]. Thus, aspirin use was not included in our analyses.

In conclusion, data from the WHI OS show that coffee consumption is related to a slightly higher incidence of colorectal cancer. Existing evidence has been inconclusive, and the current results add further to the conflicting data. Data showing that using a nondrip brew method for preparing coffee may elevate the risk of developing colorectal cancers appears to be a new finding that contradicts theories put forward on other manuscripts and should be explored further.

## Supplementary Material

The Supplementary Materials consist of four tables displaying additional data from analyses (Appendix Table A-D). Appendix Table A displays results of the analysis of the relationship between decaf coffee and colorectal cancer incidence. Appendix Table B displays results of the analysis of the relationship between total coffee consumption (decaf and regular coffee) and colorectal cancer incidence. Appendix Table C displays the full multivariable model including coffee consumption, covariates, and incidence of colorectal cancer. Appendix Table D displays the results of the analysis of subgroup effects for important covariates.

## Figures and Tables

**Table 1:** Demographic and physiologic characteristics by cups of regular coffee consumption (*n* = 83972).

Variable	None (*n* = 37082)		>0–4 cups/day (*n* = 38867)		4+ cups/day (*n* = 8023)		Trend *p* value^1^
	*n*	%	*n*	%	*n*	%	
Age, mean (SD)	63.5 (7.4)		63.7 (7.3)		62.8 (7.0)		<0.001
Ethnicity							<0.001^2^
White	30570	82.4	33136	85.3	7502	93.5	
African American	3641	9.8	2286	5.9	149	1.9	
Hispanic	1066	2.9	1587	4.1	184	2.3	
American Indian	143	0.4	161	0.4	33	0.4	
Asian	1162	3.1	1165	3.0	70	0.9	
Other/unknown	500	1.3	532	1.4	85	1.1	
Education							<0.001^3^
≤high school/GED	7525	20.3	8006	20.6	1858	23.2	
School after high school	13170	35.5	14318	36.8	3075	38.3	
College degree or higher	16387	44.2	16543	42.6	3090	38.5	
Alcohol consumption							<0.001^3^
Non-/past drinker	13329	35.9	9290	23.9	1897	23.6	
<1 drink/day	20361	54.9	23432	60.3	4798	59.8	
≥1 drink/day	3392	9.1	6145	15.8	1328	16.6	
Smoking^4^							<0.001^5^
Never	21071	56.8	18592	47.8	2917	36.4	
Past, <20 pack-years	9354	25.2	10762	27.7	1929	24.0	
Past, ≥20 pack-years	4564	12.3	6146	15.8	1656	20.6	
Current, <20 pack-years	575	1.6	1034	2.7	364	4.5	
Current, ≥20 pack-years	755	2.0	1412	3.6	966	12.0	
BMI (kg/m^2^), mean (SD)	27.3 (6.1)		27.1 (5.7)		27.1 (5.6)		<0.001
<25	15370	41.4	15975	41.1	3200	39.9	
25–30	12161	32.8	13553	34.9	2877	35.9	
>30	9551	25.8	9339	24.0	1946	24.3	
Physical activity (MET-hr/wk), mean (SD)	14.2 (14.6)		13.6 (14.1)		13.0 (14.0)		<0.001
Energy intake (kcal/day), mean (SD)	1547.1 (592.2)		1573.0 (592.9)		1674.0 (636.8)		<0.001
Red meat intake (oz.), mean (SD)	1.3 (1.2)		1.4 (1.2)		1.7 (1.4)		<0.001
Fruit/vegetable intake (cups), mean (SD)	3.3 (1.5)		3.1 (1.4)		3.0 (1.5)		
Fiber intake (g), mean (SD)	16.8 (7.1)		16.3 (6.9)		16.5 (6.9)		<0.001
Calcium intake (diet + supplements), mean (SD)	1273.5 (820.8)		1207.1 (738.4)		1217.9 (720.2)		<0.001
Percentage fat intake, mean (SD)	29.3 (8.5)		30.5 (8.3)		32.1 (8.8)		<0.001
<30	20858	56.2	19676	50.6	3434	42.8	
30–40	11937	32.2	13851	35.6	2990	37.3	
>40	4287	11.6	5340	13.7	1599	19.9	
Menopausal hormone use							0.003^6^
Never	14804	39.9	15159	39.0	3509	43.7	
Past	5537	14.9	5753	14.8	1206	15.0	
Current, E-Alone	9535	25.7	9866	25.4	1661	20.7	
Current, E + P	7206	19.4	8089	20.8	1647	20.5	
NSAID use	12723	34.3	13639	35.1	2996	37.3	<0.001
History of treated diabetes	1702	4.6	1403	3.6	226	2.8	<0.001
Family history of Colorectal Cancer							0.61^7^
No	28391	76.6	29764	76.6	6090	75.9	
Yes	5624	15.2	5937	15.3	1226	15.3	
Missing	3067	8.3	3166	8.1	707	8.8	

^1^Trend *p* values based on either a linear regression (continuous variables) or a logistic (dichotomous variables) with the variable of interest as a function of linear coffee level (None = 1, >0–4 = 2, and ≥4 = 3).

^2^ *p* value compares White versus non-White.

^3^ *p* values use ordinal form of variable of interest.

^4^1842 past and 33 current smokers have missing pack-year data.

^5^ *p* value compares current versus never/past smokers.

^6^ *p* value compares current versus never/past HT use.

^7^Trend test does not include participants with missing family history of colorectal cancer.

**Table 2:** Proportional Hazards analysis of the relationship between regular coffee consumption and incident colorectal cancer.

Cancer site	Level	Events	Ann rate	Multivariate adjusted^1^	
				HR (95% CI)	Trend *p* value^2^
All colorectal	None	513	0.108	1.0 (ref)	0.04
	>0–<4 cups/day	634	0.127	1.15 (1.02, 1.29)	
	4+ cups/day	135	0.129	1.14 (0.93, 1.38)	

Colon	None	438	0.092	1.0 (ref)	0.07
	>0–<4 cups/day	527	0.106	1.11 (0.98, 1.27)	
	4+ cups/day	118	0.113	1.17 (0.94, 1.44)	

Rectum	None	60	0.013	1.0 (ref)	0.53
	>0–<4 cups/day	86	0.017	1.30 (0.93, 1.82)	
	4+ cups/day	14	0.013	0.95 (0.52, 1.72)	

Rectosigmoid	None	27	0.006	1.00 (ref)	0.40
	>0–<4 cups/day	33	0.007	1.15 (0.69, 1.94)	
	4+ cups/day	9	0.009	1.39 (0.63, 3.07)	

^1^Adjusted for age, ethnicity, education, alcohol, smoking/pack-years, BMI, physical activity, energy intake, red meat intake, fruit/vegetable intake, percent calories from fat, fiber intake, calcium intake, hormone use, NSAID use, history of treated diabetes, and family history of colorectal cancer.

^2^Trend *p* value calculated from a separate model with the outcome of interest as a function of linear coffee level (None = 1, >0–4 = 2, ≥4 = 3).
