# Supplementary material for: Coffee Consumption and the Incidence of Colorectal Cancer in Women
Source: J Cancer Epidemiol. 2016 Apr 28;2016:6918431. doi: 10.1155/2016/6918431 (PMC4864536; doi:10.1155/2016/6918431)
Supplement: Supplementary file 1 — The Supplementary Materials consist of four tables displaying additional data from analyses (Appendix Table A-D). Appendix Table A displays results of the analysis of the relationship between decaf coffee and colorectal cancer incidence. Appendix Table B displays results of the analysis of the relationship between total coffee consumption (decaf and regular coffee) and colorectal cancer incidence. Appendix Table C displays the full multivariable model including coffee consumption, covariates, and incidence of colorectal cancer. Appendix Table D displays the results of the analysis of subgroup effects for important covariates. [file 6918431.f1.docx]

Appendix Table A. Proportional Hazards analysis of the relationship between decaf coffee consumption and incident colorectal cancer.

| Cancer Site | Level |  |  | Unadjusted^1^ | | Multivariate Adjusted^1,2^ | |
| --- | --- | --- | --- | --- | --- | --- | --- |
|  |  | **Events** | **Ann Rate** | **HR (95% CI)** | **p-value** | **HR (95% CI)** | **p-value** |
| All Colorectal | None | 877 | 0.119 | 1.00 (ref) | 0.78 | 1.00 (ref) | 0.95 |
|  | >0 - <4 cups / day | 360 | 0.117 | 1.01 (0.89, 1.14) |  | 0.99 (0.88, 1.12) |  |
|  | 4+ cups / day | 44 | 0.123 | 1.06 (0.79, 1.44) |  | 1.02 (0.75, 1.38) |  |
| Colon | None | 732 | 0.100 | 1.00 (ref) | 0.42 | 1.00 (ref) | 0.69 |
|  | >0 - <4 cups / day | 312 | 0.102 | 1.04 (0.92, 1.19) |  | 1.02 (0.89, 1.17) |  |
|  | 4+ cups / day | 38 | 0.106 | 1.10 (0.80, 1.53) |  | 1.05 (0.76, 1.45) |  |
| Rectum | None | 115 | 0.016 | 1.00 (ref) | 0.53 | 1.00 (ref) | 0.50 |
|  | >0 - <4 cups / day | 39 | 0.013 | 0.83 (0.58, 1.19) |  | 0.83 (0.58, 1.20) |  |
|  | 4+ cups / day | 6 | 0.017 | 1.10 (0.49, 2.51) |  | 1.05 (0.46, 2.39) |  |
| Rectosigmoid | None | 54 | 0.007 | 1.00 (ref) | 0.09 | 1.00 (ref) | 0.13 |
|  | >0 - <4 cups / day | 14 | 0.005 | 0.64 (0.36, 1.15) |  | 0.68 (0.38, 1.23) |  |
|  | 4+ cups / day | 1 | 0.003 | 0.40 (0.06, 2.88) |  | 0.40 (0.06, 2.89) |  |

^1^All models are adjusted for enrollment in WHI extension periods

^2^Adjusted for age, ethnicity, education, alcohol, smoking/pack years, BMI, physical activity, energy intake, red meat intake, fruit/vegetable intake, percent calories from fat, fiber intake, calcium intake, hormone use, NSAID use, history of treated diabetes, and family history of colorectal cancer

^3^Trend p-value calculated from a separate model with the outcome of interest as a function of linear coffee level (None=1, >0-4=2, ≥4=3)

Appendix Table B. Proportional Hazards analysis of the relationship between total coffee consumption and incident colorectal cancer.

| Cancer Site | Level |  |  | Unadjusted^1^ | | Multivariate Adjusted^1,2^ | |
| --- | --- | --- | --- | --- | --- | --- | --- |
|  |  | **Events** | **Ann Rate** | **HR (95% CI)** | **p-value** | **HR (95% CI)** | **p-value** |
| All Colorectal | None | 321 | 0.104 | 1.00 (ref) | 0.004 | 1.00 (ref) | 0.07 |
|  | >0 - <4 cups / day | 746 | 0.124 | 1.19 (1.05, 1.36) |  | 1.12 (0.98, 1.28) |  |
|  | 4+ cups / day | 215 | 0.129 | 1.27 (1.06, 1.50) |  | 1.17 (0.97, 1.40) |  |
| Colon | None | 272 | 0.088 | 1.00 (ref) | 0.004 | 1.00 (ref) | 0.07 |
|  | >0 - <4 cups / day | 624 | 0.103 | 1.18 (1.02, 1.36) |  | 1.10 (0.95, 1.27) |  |
|  | 4+ cups / day | 187 | 0.112 | 1.30 (1.08, 1.57) |  | 1.19 (0.98, 1.45) |  |
| Rectum | None | 39 | 0.013 | 1.00 (ref) | 0.50 | 1.00 (ref) | 0.88 |
|  | >0 - <4 cups / day | 98 | 0.016 | 1.29 (0.89, 1.87) |  | 1.20 (0.82, 1.76) |  |
|  | 4+ cups / day | 23 | 0.014 | 1.11 (0.66, 1.86) |  | 0.98 (0.58, 1.67) |  |
| Rectosigmoid | None | 19 | 0.006 | 1.00 (ref) | 0.52 | 1.00 (ref) | 0.66 |
|  | >0 - <4 cups / day | 37 | 0.006 | 1.00 (0.58, 1.74) |  | 0.99 (0.56, 1.74) |  |
|  | 4+ cups / day | 13 | 0.008 | 1.30 (0.64, 2.64) |  | 1.21 (0.58, 2.56) |  |

^1^All models are adjusted for enrollment in WHI extension periods

^2^Adjusted for age, ethnicity, education, alcohol, smoking/pack years, BMI, physical activity, energy intake, red meat intake, fruit/vegetable intake, percent calories from fat, fiber intake, calcium intake, hormone use, NSAID use, history of treated diabetes, and family history of colorectal cancer

^3^Trend p-value calculated from a separate model with the outcome of interest as a function of linear coffee level (None=1, >0-4=2, ≥4=3)

Appendix Table C. Colorectal Cancer by Regular Coffee Consumption^1^ (3-level) Full Model Results

| Variable | Level | HR (95% CI) | p-value |
| --- | --- | --- | --- |
| Coffee |  |  | 0.07 |
|  | None | 1.0 (ref) |  |
|  | >0 - <4 cups / day | 1.15 (1.02, 1.29) |  |
|  | 4+ cups / day | 1.14 (0.93, 1.38) |  |
| Age (5-year increase) |  | 1.32 (1.26, 1.37) | <0.001 |
| Ethnicity |  |  | 0.29 |
|  | White (ref) | 1.00 (ref) |  |
|  | African American | 1.12 (0.90, 1.39) |  |
|  | Asian | 0.81 (0.55, 1.21) |  |
|  | Hispanic | 0.84 (0.58, 1.22) |  |
|  | Other/Unknown | 0.72 (0.44, 1.17) |  |
| Education |  |  | 0.01 |
|  | ≤ High school / GED | 1.00 (ref) |  |
|  | School after high school | 1.26 (1.08, 1.47) |  |
|  | College degree or higher | 1.23 (1.05, 1.44) |  |
| Physical Activity (5 MET-hr/wk increase) |  | 0.98 (0.96, 1.01) | 0.13 |
| Alcohol |  |  | 0.59 |
|  | Never/Past | 1.00 (ref) |  |
|  | <1 drink/day | 1.07 (0.94, 1.22) |  |
|  | ≥1 drink/day | 1.02 (0.83, 1.25) |  |
| Smoking^2^ |  |  | <0.001 |
|  | Never | 1.00 (ref) |  |
|  | Past, <20 pack-years | 1.00 (0.87, 1.14) |  |
|  | Past, ≥20 pack-years | 1.34 (1.15, 1.56) |  |
|  | Current, <20 pack-years | 1.05 (0.70, 1.58) |  |
|  | Current, ≥20 pack-years | 1.63 (1.26, 2.11) |  |
| Body Mass Index (5 kg/m^2^ increase) |  | 1.10 (1.05, 1.15) | <.001 |
| Total Energy Intake (500kcal/day increase) |  | 0.99 (0.91, 1.08) | 0.87 |
| Red Meat Intake (1 oz increase) |  | 1.03 (0.97, 1.08) | 0.34 |
| Fruit / Vegetable Intake (1 cup increase) |  | 0.98 (0.92, 1.04) | 0.42 |
| Fiber Intake (10g/day increase) |  | 1.05 (0.89, 1.23) | 0.58 |
| Calcium Intake (500 mg/day increase) |  | 1.00 (0.96, 1.04) | 0.93 |
| Percent Caloric Intake from Fat (5 percent increase) |  | 1.02 (0.98, 1.07) | 0.31 |
| Hormone Use |  |  | <0.001 |
|  | Never | 1.00 (ref) |  |
|  | Past | 0.89 (0.76, 1.04) |  |
|  | Current, E-Alone | 0.72 (0.62, 0.84) |  |
|  | Current, E+P | 0.75 (0.64, 0.89) |  |
| NSAID use |  | 0.87 (0.77, 0.97) | 0.02 |
| History of treated diabetes |  | 1.27 (0.99, 1.63) | 0.06 |
| Family History of Colorectal Cancer^3^ |  |  | 0.003 |
|  | No | 1.0 (ref) |  |
|  | Yes | 1.24 (1.08, 1.43) |  |

^1^Model additionally adjusted for enrollment in WHI extension periods

^2^ Smoking has an additional categorical level indicating missing pack-years data. P-value based on contrast of known responses.

^3^Family history of colorectal cancer has an additional categorical level indicating missing data. p-value based on contrast of ‘Yes’ vs. ‘No’ responses.

Appendix Table D. Coffee Consumption on Colorectal Cancer by Subgroups

|  |  | Cups of Regular Coffee per Day | | | | |  |
| --- | --- | --- | --- | --- | --- | --- | --- |
|  |  | ***Events (Ann %)*** | | | ***HR (95% CI0 vs. 0*** | | ***Interaction*** |
| *Variable* | ***Level*** | ***0*** | ***>0 - <4*** | ***≥ 4*** | ***>0 - <4*** | ***≥ 4*** | ***trend p-value^1^*** |
| All Participants |  | 513 (0.11) | 634 (0.13) | 135 (0.13) | 1.15 (1.02, 1.29) | 1.14 (0.93, 1.38) |  |
| Age |  |  |  |  |  |  | 0.11^2^ |
|  | 50 – 59 | 83 (0.05) | 105 (0.06) | 34 (0.09) | 1.25 (0.94, 1.67) | 1.63 (1.09, 2.44) |  |
|  | 60 – 69 | 236 (0.11) | 294 (0.13) | 68 (0.14) | 1.14 (0.96, 1.35) | 1.10 (0.84, 1.45) |  |
|  | 70 – 79 | 194 (0.19) | 235 (0.22) | 33 (0.19) | 1.12 (0.92, 1.35) | 0.89 (0.61, 1.29) |  |
| Ethnicity |  |  |  |  |  |  | 0.89 |
|  | White | 430 (0.11) | 554 (0.13) | 126 (0.13) | 1.15 (1.01, 1.31) | 1.12 (0.92, 1.38) |  |
|  | African American | 53 (0.13) | 44 (0.18) | 4 (0.24) | 1.25 (0.84, 1.87) | 1.53 (0.55, 4.24) |  |
|  | Hispanic | 13 (0.11) | 13 (0.08) | 3 (0.15) | 0.67 (0.31, 1.45) | 1.23 (0.35, 4.32) |  |
|  | Asian | 11 (0.09) | 14 (0.11) | 1 (0.12) | 1.20 (0.55, 2.65) | 1.35 (0.17, 10.47) |  |
|  | Other / Unknown | 6 (0.08) | 9 (0.11) | 1 (0.07) | 1.35 (0.48, 3.78) | 0.79 (0.10, 6.53) |  |
| Alcohol Use |  |  |  |  |  |  | 0.43^3^ |
|  | No | 186(0.12) | 157 (0.14) | 29 (0.13) | 1.17 (0.95, 1.45) | 1.03 (0.69, 1.53) |  |
|  | <1 drink/day | 285 (0.11) | 382 (0.12) | 77 (0.12) | 1.13 (0.97, 1.31) | 1.05 (0.82, 1.36) |  |
|  | ≥ 1 drink/day | 42 (0.09) | 95 (0.11) | 29 (0.16) | 1.24 (0.86, 1.79) | 1.70 (1.06, 2.74) |  |
| Smoking |  |  |  |  |  |  | 0.61 |
|  | Never | 279 (0.10) | 297 (0.12) | 39 (0.10) | 1.17 (0.99, 1.38) | 0.97 (0.69, 1.36) |  |
|  | Past, <20 pack-years | 119 (0.10) | 154 (0.11) | 30 (0.12) | 1.14 (0.90, 1.45) | 1.24 (0.83, 1.86) |  |
|  | Past, ≥20 pack-years | 83 (0.15) | 128 (0.17) | 31 (0.15) | 1.15 (0.87, 1.52) | 1.07 (0.71, 1.62) |  |
|  | Current, <20 pack-years | 4 (0.06) | 12 (0.09) | 9 (0.20) | 1.63 (0.53, 5.06) | 3.69 (1.14, 12.01) |  |
|  | Current, ≥20 pack-years | 15 (0.18) | 32 (0.21) | 22 (0.20) | 1.12 (0.61, 2.07) | 1.16 (0.60, 2.23) |  |
| Current HT Use |  |  |  |  |  |  | 0.52 |
|  | No | 335 (0.13) | 409 (0.16) | 99 (0.17) | 1.15 (0.99, 1.33) | 1.21 (0.96, 1.53) |  |
|  | Yes | 178 (0.08) | 225 (0.09) | 36 (0.08) | 1.15 (0.94, 1.40) | 0.98 (0.68, 1.41) |  |
| Current HT Type^4^ |  |  |  |  |  |  | 0.49 |
|  | E-Alone | 111 (0.09) | 121 (0.09) | 20 (0.09) | 1.00 (0.77, 1.30) | 0.99 (0.61, 1.61) |  |
|  | E+P | 67 (0.07) | 104 (0.09) | 16 (0.07) | 1.33 (0.98, 1.81) | 0.99 (0.57, 1.72) |  |
| BMI |  |  |  |  |  |  | 0.39^2^ |
|  | Normal | 180 (0.09) | 226 (0.11) | 51 (0.12) | 1.17 (0.96, 1.43) | 1.26 (0.92, 1.73) |  |
|  | Overweight | 188 (0.12) | 217 (0.12) | 43 (0.11) | 1.00 (0.82, 1.22) | 0.91 (0.65, 1.27) |  |
|  | Obese | 145 (0.13) | 191 (0.17) | 41 (0.17) | 1.31 (1.05, 1.63) | 1.28 (0.90, 1.82) |  |
| Coffee Type^4^ |  |  |  |  |  |  | 0.06 |
|  | Non-drip | 513 (0.11) | 172 (0.13) | 35 (0.18) | 1.05 (0.89, 1.25) | 1.43 (1.01, 2.02) |  |
|  | Drip |  | 457 (0.13) | 100 (0.12) | 1.20 (1.05, 1.36) | 1.08 (0.86, 1.34) |  |

^1^Interaction p-values were calculated in a separate model with colorectal cancer as a function of the subgroup of interest, linear coffee level (0 cups=1, >0-<4 cups=2, ≥4 cups=3) and their interaction, with adjustment for all variables in Table 3.

^2^Interactions for continuous markers were evaluated with the interaction between coffee level by the continuous version of the subgroup of interest

^3^interaction tests linear alcohol trend (1=”No”, 2=”<1 drink/day”, 3=”≥1 drink/day” by linear coffee level

^3^Among participants reporting current HT at baseline only

^4^Both coffee type subgroups share a reference group of participants with no regular coffee consumption
